# Low self esteem contributes to medical student social media addiction through chain mediation by academic Involution and anxiety

**DOI:** 10.1038/s41598-025-24317-9

**Published:** 2025-11-18

**Authors:** Xiaodi Ma, Bing Li, Hong Nie, Wenlong Ding, Ruirui He, Wanyu He, Wanting Liu

**Affiliations:** 1 Heilongjiang University of Traditional Chinese Medicine, Harbin, 150040 Heilongjiang China; 2 Heilongjiang University of Traditional Chinese Medicine, Harbin, 150040 Heilongjiang China; 3 Yichun Vocational College, Yinchun, 153000 Heilongjiang China; 4 Shaanxi Provincial Hospital of Traditional Chinese Medicine, Xi’an, 710003 Shaanxi China; 5 Heilongjiang Academy of Traditional Chinese Medicine, Harbin, 150036 Heilongjiang China

**Keywords:** Self-esteem, Social media addiction, Anxiety, Academic over-competition (involution), Medical students, Psychology, Health occupations, Medical research

## Abstract

This study aimed to investigate the impact of low self-esteem on social media addiction among medical students, as well as the underlying mechanisms involving academic involution and anxiety. A cross-sectional survey was conducted among 1055 medical students from two universities in China. Subjective data regarding self-esteem, academic involution, anxiety, and social media addiction were collected. Correlation analyses were performed, and a mediation model was constructed to examine the relationships. The results indicated that: (1) self-esteem, academic involution, anxiety, and social media addiction were significantly correlated with each other; (2) self-esteem significantly negatively predicted social media addiction; (3) in the relationship between self-esteem and social media addiction, anxiety and academic involution played both independent mediating roles and a sequential mediating role. Specifically, self-esteem not only directly influenced social media addiction but also exerted an indirect effect through the chain mediation of academic involution and anxiety. These findings provide new insights into the mechanisms underlying social media addiction among medical students and suggest potential targets for prevention and intervention.

## Introduction

Social media addiction has emerged as a common global issue. It refers to the situation where individuals overly focus on social media, invest a substantial amount of time and energy, and have difficulty self - regulating this usage behavior, which exerts negative impacts on other important aspects of an individual’s life^1^. A meta - analysis revealed that the prevalence of social media addiction among college students worldwide was approximately 18.4%. Notably, the prevalence in the Asian region was significantly higher than that in other regions, amounting to 24.3%^2^. Medical students, constituting a distinct subset within the college - student population, are confronted with the compounded stressors stemming from both academic pursuits and clinical practice. This unique situation renders them more vulnerable to the risk of social media addiction compared to their non - medical counterparts^3,4^. Epidemiological investigations have revealed that the prevalence of social media addiction among medical students is 30.1%, a rate significantly higher than that observed in other populations^5^. Studies have demonstrated that social media addiction is not only associated with academic performance decline and social skill impairment among medical students, but may also elevate their risk of chronic diseases and precipitate negative psychological outcomes such as anxiety and depression^6^. A profound exploration of the influencing factors and underlying mechanisms of social media addiction among medical students holds substantial significance.

Self - esteem, which mirrors the extent to which an individual acknowledges themselves and perceives their self - worth, stands as a fundamental and core characteristic within the realm of mental health^7^. Self - esteem serves as a fundamental cornerstone in the construction of the relationship between an individual and the social environment. It plays a pivotal role in how an individual interacts with and perceives the surrounding social context^8–11^. In the context of Beck’s cognitive - behavioral theory, the dysfunctional cognitive schema, through the induction of persistent negative affective states, impels individuals to adopt social media use as a strategy for regulating negative emotions. This reinforcing cycle is closely associated with social media addiction^12^. A recent study has revealed that there exists a significant negative correlation between self - esteem levels and the incidence of social media addiction in the population of medical students^13^. A comprehensive large - scale meta - analysis, which integrated data from multiple relevant studies, has demonstrated that the percentage of individuals with low self - esteem who exhibit dependency on social media is significantly higher compared to that of those with high self - esteem^14^. Findings of the survey demonstrate that medical students are confronted with intense academic pressure. This pressure has the potential to exacerbate the fluctuations in self - esteem. Specifically, medical students with low self - esteem are more prone to utilize social media as a stress - alleviation tool within a high - pressure context^15^. In light of the above - mentioned background and considerations, this research puts forward Hypothesis 1: A significant negative correlation exists between self - esteem and social media addiction in the medical student population.

According to social comparison theory, individuals with low self-esteem struggle to objectively assess their own abilities and worth, and tend to engage in upward social comparisons, viewing external indicators such as academic achievement as core criteria of self-value^16^. This cognitive pattern, reliant on external validation, makes them highly susceptible to being drawn into a state of irrational competition, known as “academic involution”^17^. Originally derived from anthropological studies of agricultural economic models, “involution” describes a phenomenon within resource-limited systems where continuous excessive input of labor leads to diminishing marginal returns, essentially representing “growth without development"^18^. This concept has since been extended to the field of education, describing behavioral patterns among students characterized by excessive input, maladaptive competition, and continuously diminishing learning returns^19^. Research indicates that students with low self-esteem, due to lower self-efficacy, often perceive themselves as lacking ability and thus are more inclined to engage in competition by indefinitely prolonging study time^20^. In such a long-term competitive and involutionary environment, negative emotions such as anxiety and depression are easily triggered^21^. To escape this psychological discomfort, individuals may turn to social media as an emotional regulation tool, ultimately increasing the risk of social media addiction^22^;^23^. Recent studies on involution and social media addiction have found a significant relationship between the two^24^. Thus, research hypothesis 2 is proposed: Academic involution mediates the relationship between self-esteem and social media addiction.

Anxiety is a multifaceted and ubiquitous emotional state. It is principally characterized by the manifestation of a constellation of negative emotions, including but not limited to restlessness, nervousness, apprehension, and fear^25^. According to cognitive theory, individuals with low self-esteem exhibit pronounced cognitive dysregulation. This cognitive dysregulation leads to the emergence of cognitive biases. Cognitive biases, in turn, serve as a catalyst for the development of anxiety^12^. Neuroimaging studies have shown that in individuals with cognitive dysfunction, the activation intensity of the amygdala is 1.7 times higher than that of the healthy control group, and the cognitive function of the dorsolateral prefrontal cortex is impaired, resulting in a 38% increase in their anxiety^26^. Empirical evidence has shown that college students with low self-esteem have higher levels of stress hormones when dealing with difficult tasks and are more prone to anxiety^27,28^.Anxiety is an important predictor of social media addiction among medical students, and there is a positive correlation between the two^29^. According to Gross’s emotion regulation theory, anxiety substantially impairs the accurate perception of positive emotions. This impairment reduces the ability to experience satisfaction in daily life, compelling individuals to turn to social media as a compensatory mechanism for positive affirmation. This behavior, in turn, elevates the likelihood of developing social media addiction^30^. Thus, research hypothesis 3 is proposed: Anxiety plays a mediating role between self - esteem and social media addiction.

Involution and anxiety are likely to serve as mediating variables in the relationship between self - esteem and social media addiction among medical students, and a certain degree of correlation exists between them.The transactional model of stress posits that the unrelenting competitive pressure of involution among medical students compromises their emotional regulation capabilities. This, in turn, gives rise to negative emotions, prominently including anxiety^31^. Empirical investigations have revealed a significant correlation between involution and anxiety. In the context of involution, individuals are confronted with incessant and highly intensive learning processes. This unceasing academic or work - related pressure stemming from involution forces individuals to remain in a state of perpetual tension. As a result, their psychological stress accumulates, and the level of anxiety escalates^32^. On the contrary, a decrease in the intensity of involution can substantially mitigate the anxiety levels among medical students^33^. Consequently, the present research postulates Hypothesis 4: Involution and anxiety jointly exert a chain - mediating effect in the relationship between self - esteem and social media addiction.

**Fig. 1:**
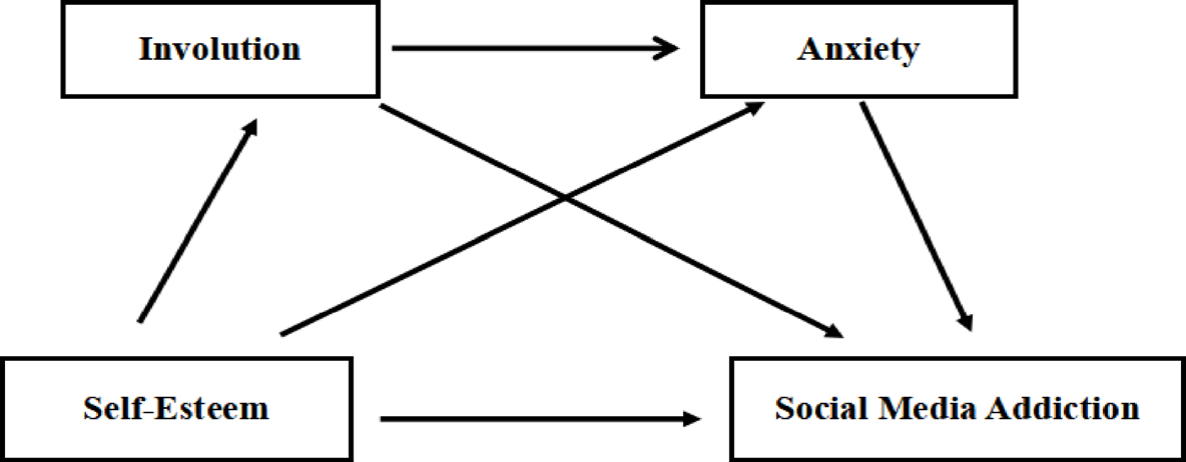
Hypothetical chain mediation model where involution and anxiety mediate the relationship between self-esteem and social media addiction.

In conclusion, the present research is dedicated to examining the relationship between self - esteem and social media addiction among medical students. Therefore, this study constructs a hypothetical model diagram (Fig. 1) to further explore the potential mechanisms underlying the relationship between self-esteem and social media addiction among medical students.

## Methods

### Procedure and participants

This cross-sectional study was conducted during the winter semester of 2024 among medical students from two public universities in Heilongjiang Province, China. A convenience sampling strategy was adopted, distributing the survey electronically to students by class. Prior to data collection, researchers obtained permission and support from the university authorities and class advisors, who helped introduce the study to potential participants. The informed consent process was implemented digitally via an online consent form presented on the first page of the questionnaire. This form clearly outlined the study purpose, ensuring respondent anonymity, the confidentiality of data, the voluntary nature of participation, and the intended use of the data. Participants could only proceed to the formal questionnaire section after reading and selecting “Agree”; those who selected “Disagree” were automatically directed to the end of the survey. To enhance data quality and reduce response bias, several strategies were employed in the questionnaire design. These included using different scale formats and anchor points, randomizing or logically grouping the order of items, and incorporating instructions that emphasized there were no right or wrong answers while encouraging truthful responses. Completing the entire questionnaire took approximately 10 min. The study received ethical approval from the institutional medical ethics committee prior to its commencement and was conducted in accordance with the Declaration of Helsinki. A total of 1313 students initially completed the survey. After excluding responses with excessively short completion times or patterned answering, 1055 valid datasets were retained for final analysis (see Table 1).

**Table 1:** Basic information of the participants.

Items		*N*	Percent
Gender	Male	240	22.7%
	Female	815	77.3%
Father’s degree	Unascertained	72	6.8%
	Primary education level and below	191	18.1%
	Secondary education level	684	64.8%
	Tertiary education	102	9.7%
	Postgraduate degree	6	0.6%
Mother’s degree	Unspecified	68	6.4%
	Primary education level and below	228	21.6%
	Secondary education level	650	61.6%
	Tertiary education	104	9.9%
	Postgraduate degree	5	0.5%
Grade	First-year undergraduate	482	45.7%
	Second-year undergraduate	485	46.0%
	Third-year undergraduate	81	7.7%
	Fourth-year undergraduate	2	0.2%
Situation of only children	Only-child status	414	39.2%
	Non-only-child status	641	60.8%

### Measurement tools

Due to the heavy academic tasks and tight course schedules of medical students, before the questionnaire filling, the investigators elaborated and defined the structural information covering the main variables in detail and clearly. In view of this, when selecting the research measurement tools, there was a strong preference for relatively concise tools. This could effectively reduce the cognitive load of medical students when participating in the survey, cut down their time investment, and at the same time, maximize the avoidance of misunderstandings or biases to ensure the accuracy and effectiveness of the survey results^34,35^.

#### Self-esteem

One question was used to assess the self - esteem level of the sample in this study. Question: Do you feel that your abilities are insufficient, that you are inferior to others in life and study, and that when you disagree with others, you subconsciously think that you are wrong? This question adopts a 5 - point scale, with scores ranging from 1 (completely inconsistent) to 5 (completely consistent). The higher the score, the lower the individual’s self - esteem level. This has been found to have good robustness in previous studies. This has been widely used in previous studies^36^.

#### Involution

The Involution Perception Measurement Questionnaire developed by Zhang Wen et al.^16^. was used for assessment. This questionnaire was developed based on China’s social and cultural context and consists of 18 items grouped into four dimensions: psychological pressure, social norms, competitive behavior, and resource scarcity. After multiple reliability and validity tests, it has demonstrated good applicability among domestic populations^37^. The scale adopts a 7-point Likert scale, with each item scored from 1 (completely inconsistent) to 7 (completely consistent), yielding a total score range of 18–126. The higher the total score, the higher the degree of involution perceived by the individual. The original study reported a Cronbach’s alpha coefficient of 0.75^16^.

#### Anxiety

The 2 - item Generalized Anxiety Disorder Scale (GAD − 2) was used to assess the anxiety levels of the sample in this study^38,39^ .Translated and revised into Chinese by Qian Jie, it has demonstrated good applicability in domestic populations^40^. The GAD − 2 consists of two items: (1) Feeling nervous, anxious, or on edge; (2) Not being able to stop or control worry. It adopts a 4 - point Likert scale, with scores ranging from 1 (never) to 4 (almost every day), and the total score range is 2–8. The higher the score, the higher the degree of anxiety perceived by the individual. The original study reported a Cronbach’s alpha coefficient of 0.80^40^.

#### Social media addiction

The Bergen Social Media Addiction Scale developed by Andreassen et al.^41^. Localized by Jiang Yongzhi, it has proven to be well-suited for domestic groups^42^. This scale contains 6 items and adopts a 5 - point Likert scale. The scoring range of each question is from 1 (very rarely) to 5 (always). The total score ranges from 6 to 30. The higher the total score, the more severe the social media addiction. The original study reported a Cronbach’s alpha coefficient of 0.87^42^.

### Statistical analyses

Data analyses were conducted using IBM SPSS Statistics (Version 26.0). First, we performed Pearson correlation analyses to examine the relationships among the main variables. Second, to test the hypothesized chain mediation model (self-esteem → academic involution → anxiety → social media addiction), we used Model 6 of the PROCESS macro for SPSS (version 4.0)^43^. The analysis utilized a bias-corrected bootstrapping method with 5,000 samples to generate 95% confidence intervals (CIs) for the indirect effects. An effect was considered statistically significant if the 95% CI did not include zero. Demographic variables (gender, age, grade, parental education, and only-child status) were included as covariates. The significance level was set at α = 0.05.

## Results

### Common method bias and reliability

The results of the common method bias test in this study found that there were 6 factors with eigenvalues greater than 1. The first factor accounted for 22.71% of the total variance, which was less than the threshold of 40%^44^, indicating that there was no obvious risk of common method bias in this study. Additionally, a reliability analysis was conducted on the collected sample data. The results indicated that all scales demonstrated good internal consistency in this survey, with Cronbach’s alpha coefficients ranging from 0.80 to 0.88.

### Correlation analysis

The results in Table 2 show that self - esteem has a significant negative correlation with social media addiction (*r* = − 0.233, *P* < 0.01), involution (*r* = − 0.257, *P* < 0.01), and anxiety (*r* = − 0.327, *P* < 0.01). Social media addiction has a significant positive correlation with involution (*r* = 0.303, *P* < 0.01) and anxiety (*r* = 0.332, *P* < 0.01). Involution has a significant positive correlation with anxiety (*r* = 0.360, *P* < 0.01) (see Table 2).

**Table 2:** Correlational analyses.

Variables	M	SD	1	2	3	4	5	6
1. Age	–	–	–					
2. Situation of only children	–	–	−0.038	–				
3. Self-esteem	2.33	1.00	−0.003	−0.015	–			
4. Social media addiction	15.20	4.41	0.034	0.06	−0.233**	–		
5. Involution	68.44	12.07	0.026	0.000	−0.257**	0.303**	–	
6. Anxiety	3.17	1.33	−0.019	−0.034	−0.327**	0.332**	0.360**	–

*p<0.05; **p<0.01; ***p<0.001.

### Mediation model test

**Fig. 2:**
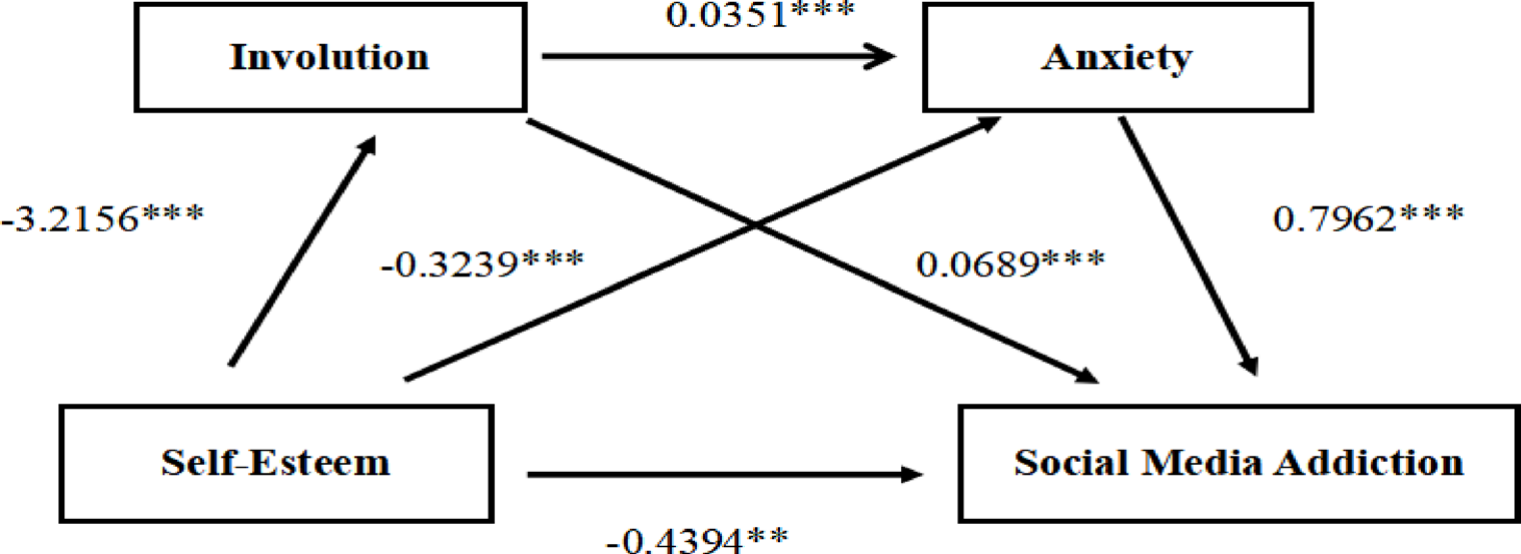
A chain-mediated model of self-esteem and social media addiction between involution and anxiety.

After controlling for demographic variables (gender, age, grade, parents’ education level, and only-child status), the results of Tables 2 and 3, as well as Fig. 2, indicate that: self - esteem had a significant negative direct predictive effect on medical students’ social media addiction (total effect: *β* = -1.0085, *P* < 0.001). When mediating variables (involution, anxiety) were added, the direct effect of self - esteem on social media addiction was still significant (*β* = -0.4394, *P* < 0.01). Self - esteem significantly negatively predicted involution (β = -3.2156, *P* < 0.001), and involution significantly positively predicted social media addiction (β = 0.0689, *P* < 0.001). Self - esteem significantly negatively predicted anxiety (*β* = -0.3239, *P* < 0.001), and anxiety significantly positively predicted social media addiction (*β* = 0.7962, *P* < 0.001). Finally, there was a chain - mediating effect of involution and anxiety between self - esteem and social media addiction (*β* = -0.0899, *P* < 0.001), forming a chain - like path.

**Table 3:** Mediation model testing.

Outcome variable	Predictor variable	β	SE	t	*R*²	F
Involution	Self-esteem	−3.2156	0.3610	−8.91***	0.0748	12.08***
Anxiety	Self-esteem	−0.3239	0.0384	−8.43***	0.2043	33.58***
	Involution	0.0351	0.0032	11.07***		
Social media addiction	Self-esteem	−0.4394	0.1340	−3.28**	0.1718	24.08***
	Involution	0.0689	0.0113	6.09***		
	Anxiety	0.7962	0.1043	7.63***		

**p<0.01; ***p<0.001.

### The chain mediation effect of self-esteem and social media addiction between Involution and anxiety

Based on correlation analysis and mediation effect testing methods proposed by relevant literature, this study conducted path analysis with gender, age, grade, parents’ education level, and only-child status as covariates, self-esteem as the independent variable (X), social media addiction as the dependent variable (Y), and involution (M1) and anxiety (M2) as mediating variables. Please refer to Fig. 2.

The research findings indicate: Total Effect of Self-esteem on Social Media Addiction: The total effect size of self-esteem on social media addiction is -1.0085, with a direct effect of -0.4394 and an effect size of 43.6% (*P* < 0.05).Mediation Effect of Involution: Involution partially mediates the relationship between self-esteem and social media addiction, with a mediation effect size of -0.2214 and an effect size of 22.0% (*P* < 0.05).Mediation Effect of Anxiety: Anxiety also partially mediates the relationship between self-esteem and social media addiction, with a mediation effect size of -0.2579 and an effect size of 25.6% (*P* < 0.05).Chain Mediation Effect: Involution and anxiety together form a chain mediation path between self-esteem and social media addiction, with a chain mediation effect size of -0.0899 and an effect size of 8.9% (*P* < 0.05).All paths’ 95% confidence intervals do not include zero, indicating that all mediation effects are significant (*P* < 0.05). Please refer to Table 4.

**Table 4:** Path analysis of the mediation model.

Paths	Effect size	Bootstrap 95% CI	Proportion of mediation effect
Total effect	−1.0085	[−1.2694,−0.7476]	100%
Direct effect	−0.4394	[−0.7022,−0.1765]	43.6%
Total indirect effect	−0.5691	[−0.7308,−0.4277]	56.4%
Ind 1	−0.2214	[−0.3400,−0.1177]	22.0%
Ind 2	−0.2579	[−0.3657,−0.1608]	25.6%
Ind 3	−0.0899	[−0.1357,−0.0534]	8.9%

## Discussion

This study explored the interrelationships among self - esteem, involution, anxiety, and medical students’ social media addiction, and constructed the internal connections among them. This study found that there were significant correlations between each pair of self - esteem, involution, anxiety, and medical students’ social media addiction. In addition, involution and anxiety served as mediating and chain - mediating roles between self - esteem and medical students’ social media addiction. These findings enriched the influencing paths of medical students’ social media addiction and provided new explanatory space for how self - esteem levels affect medical students’ social media addiction. At the same time, it also provided empirical evidence for the impacts of self - esteem, involution, and anxiety on social media addiction.

This study found that the relationship between self - esteem and medical students’ social media addiction was significant, so H1 was established. This is consistent with previous research results^45^. Based on the compensatory internet use theory, medical students with low self-esteem may struggle to attain sufficient accomplishment and belonging in real-life academic and social settings^46^. Social media provides a “compensatory environment,” offering a sense of control and self-worth that is difficult to achieve in reality^47^. This psychological compensation effect is a key mechanism underlying their dependent behavior. From a neuroendocrine perspective, Chronic low self-esteem, acting as a chronic psychological stressor, promotes addiction via an endocrine pathway: by activating the HPA axis to elevate cortisol levels, which thereby suppresses prefrontal inhibitory control and enhances reward sensitivity to online stimuli, thus establishing a vicious “stress-reward” cycle^48^. Regarding neurotransmitters, negative emotions such as anxiety and depression that often accompany low self-esteem can disrupt the balance of the dopamine and serotonin systems^49^. Dysregulation of the dopamine system increases craving for the immediate rewards provided by social media, while serotonin deficiency may worsen emotional states, creating a vicious cycle. Clinical research provides quantitative support for this: for every 1-unit increase in self-esteem, the individual’s risk of developing social media addiction decreases by 11%^50^.

In addition, this study also found that involution plays a partial mediating role between self - esteem and social media addiction of medical students, so Hypothesis 2 (H2) is supported. The results of this study are consistent with the findings of relevant meta - analysis studies^24^. From the perspectives of psychology and sociology, medical students with low self-esteem are more prone to self-doubt and negative self-evaluation in the process of social comparison. In order to verify their own values and reshape positive self-cognition, individuals tend to obtain external recognition through excessive competitive behaviors, thus falling into the “involution” pattern. Such a high-intensity competitive environment not only exacerbates their psychological pressure, but also significantly reduces their time for interaction with others in reality, unmet social needs. To make up for the lack of real-life social interaction, individuals may turn to social media to seek emotional support and a sense of belonging, thus forming an alternative social compensation mechanism^51^. Previous studies suggested that at the neuro - mechanism level, due to the long - term involution pressure, the hypothalamic - pituitary - adrenal (HPA) axis function is disordered. The increase in cortisol levels not only directly exacerbates anxiety but may also further weaken an individual’s control ability over social media use by reducing the inhibitory function of the prefrontal cortex on the amygdala^52^. A longitudinal study by Deng et al. found that there is a dose - response relationship between the salivary cortisol levels of medical students and the severity of social media addiction, suggesting that the dysregulation of the HPA axis may be a key biomarker for the transformation of involution pressure into addictive behaviors. At this time, the reward pathway is activated through the mesolimbic dopamine system, forming a stress - reward substitution mechanism, which may predict the pathological development of social media addiction^53^.

In addition, this study also found that anxiety mediates the relationship between self - esteem and social media addiction of medical students, so Hypothesis 3 (H3) is supported. The results of this study are consistent with the findings of relevant meta - analysis studies^54^. From a psychological perspective, analyzing that low self-esteem, as a psychological vulnerability factor, tends to lead individuals to generate negative evaluations of themselves, and this negative tendency provides a psychological foundation for the occurrence of anxiety^43^. A meta-analysis indicates that there is a significant positive correlation between the frequency of social media use and the level of anxiety. At the same time, it will significantly reduce individuals’ positive emotional experiences^55^. In addition, recent studies have found that when individuals with low self-esteem are exposed to content presenting an idealized self, it leads to self-evaluation bias, making them prone to falling into negative emotions such as anxiety. They often choose to escape from reality or relieve negative emotions, which in turn leads to social media addiction. Modern medicine has discovered that social media addiction is closely related to the excessive activation of the brain’s reward system. Research shows that the stimulation of dopamine release (associated with feelings of pleasure and the alleviation of negative emotions) may predict the formation of addictive behaviors^56^.

Finally, this study also found that in the influence of self - esteem on social media addiction of medical students, involution and anxiety play a chain mediating role, verifying Hypothesis 4. Learning dominates the life of medical students in college. However, medical students with low self - esteem are more likely to compare themselves with others when facing high - intensity academic competition, thus deeply perceiving the pressure of involution. This pressure makes them have a strong sense of insecurity and self - doubt at the psychological level. According to the stress - coping theory, when they are in such a high - pressure situation for a long time, anxiety will emerge. The virtual environment created by social media provides them with a haven to temporarily escape from the real - world pressure and negative emotions, making them gradually indulge in it, which may predict the development of addictive behaviors.

This research delves deep into the intricate relationships among self - esteem, involution, anxiety, and social media addiction among medical students. For the first time, it endeavors to integrate these variables, thereby extending the existing research outcomes to a certain degree. By analyzing the chain - mediating model, the study further uncovers the underlying connections and interactions among them, which holds significant theoretical and practical implications.The findings indicate that there are substantial correlations among self - esteem, involution, anxiety, and social media addiction in medical students. Notably, involution functions as a single mediator, while anxiety serves as a chain - mediator. These discoveries not only enrich the theoretical framework regarding the psychological and behavioral mechanisms between self - esteem and social media addiction in medical students but also offer novel perspectives and strategies for clinical intervention.From a theoretical perspective, this study validates the close association between self - esteem and social media addiction among medical students through empirical research. It further corroborates the adverse effects of low self - esteem on an individual’s physical and mental well - being, providing fresh evidence for understanding the pathological mechanisms of individuals with low self - esteem.

Practically, the research results imply that for the medical student population, it is crucial to prioritize the assessment and intervention of involution and anxiety to mitigate the risk of social media addiction. Clinically, this study furnishes a scientific foundation for devising preventive and intervention measures targeting the low - self - esteem phenomenon among medical students. It underscores the significance and necessity of considering the self - esteem context when treating social media addiction in medical students.Furthermore, this study recommends that subsequent research should delve deeper into the causal relationships among self - esteem, involution, and anxiety. Simultaneously, future studies should also focus on other potential variables associated with social media addiction in medical students, such as the impact of physical exercise intervention on this addictive behavior^57^. Previous studies have found that there is a negative correlation between physical exercise and social media addiction among college students^58^. Moreover, physical exercise has the potential to mitigate negative emotions among medical students, alleviate the phenomenon of involution - related behaviors, and reduce the prevalence of social media addiction within this student group. By doing so, it offers novel and innovative intervention strategies in the realm of physical and mental well - being^59^. In conclusion, this study not only enhances the understanding of the relationship between self - esteem and social media addiction among medical students, but also provides valuable theoretical and practical guidance for the maintenance and promotion of physical and mental health.

## Conclusions

This study reveals a significant correlation among self - esteem, involution, anxiety, and social media addiction of medical students. The research finds that self - esteem, involution, anxiety, and social media addiction of medical students are significantly correlated pairwise. In the influence of self - esteem on social media addiction of medical students, involution and anxiety play a separate mediating role and a chain mediating role respectively. This discovery not only provides a new theoretical basis for analyzing the formation mechanism of medical students’ social media dependence, but also establishes a key practical entry point for the formulation of targeted intervention strategies. It is recommended that subsequent research further verify the stability and universality of the action paths between variables through longitudinal research design, interdisciplinary method integration, and comparative analysis of groups with different cultural backgrounds, so as to enhance the clinical application value of the research results.

## Limitations and recommendations

This study has several limitations that should be acknowledged. First, the cross-sectional design reveals associations among variables but cannot establish causal relationships. Future research should employ longitudinal or experimental designs to verify the proposed causal pathways. Second, the sample was drawn solely from medical students at two Chinese universities, which may limit the generalizability of the findings. Subsequent studies should expand the sampling framework to include participants from diverse regions, types of institutions, and cultural backgrounds to examine the model’s cross-population stability. Third, the data were primarily collected using self-report measures, which may introduce common method bias and measurement errors due to issues such as social desirability or recall bias. Furthermore, while focusing on the mediating roles of academic involution and anxiety, this study may have overlooked other relevant variables (e.g., fear of missing out, smartphone addiction) and did not incorporate objective physiological indicators (e.g., cortisol levels). Finally, the lack of in-depth subgroup analyses based on factors such as gender or academic year may have obscured unique psychological mechanisms within specific groups (e.g., senior students facing clinical practice pressure).

Based on these limitations, future research could (1) adopt longitudinal designs to clarify causal sequences among variables; (2) expand sample sources to enhance external validity; (3) implement multi-method assessments (e.g., behavioral data, physiological indicators) to complement self-report data; (4) explore other potential mediators or moderators (e.g., fear of missing out, perceived stress) to refine the theoretical model; and (5) conduct heterogeneity analyses (e.g., by gender or academic stage) to inform tailored intervention strategies for different subgroups.

## Data Availability

The datasets used during the current study are available from the corresponding author on reasonable request.
